# Increased exogenous but unaltered endogenous carbohydrate oxidation with combined fructose-maltodextrin ingested at 120 g h^−1^ versus 90 g h^−1^ at different ratios

**DOI:** 10.1007/s00421-022-05019-w

**Published:** 2022-08-11

**Authors:** Tim Podlogar, Špela Bokal, Simon Cirnski, Gareth A. Wallis

**Affiliations:** 1 School of Sport, Exercise and Rehabilitation Sciences, College of Life and Environmental Sciences, University of Birmingham, Birmingham, UK; 2 Faculty of Health Sciences, University of Primorska, Izola, Slovenia; 3 Human Performance Centre, Ljubljana, Slovenia

**Keywords:** Exercise metabolism, Carbohydrates, Exogenous carbohydrate oxidation, Endurance exercise, Carbohydrate supplementation

## Abstract

**Purpose:**

This study aimed to investigate whether carbohydrate ingestion during 3 h long endurance exercise in highly trained cyclists at a rate of 120 g h^−1^ in 0.8:1 ratio between fructose and glucose-based carbohydrates would result in higher exogenous and lower endogenous carbohydrate oxidation rates as compared to ingestion of 90 g h^−1^ in 1:2 ratio, which is the currently recommended approach for exercise of this duration.

**Methods:**

Eleven male participants (V̇O_2peak_ 62.6 ± 7 mL kg^−1^ min^−1^, gas exchange threshold (GET) 270 ± 17 W and Respiratory compensation point 328 ± 32 W) completed the study involving 4 experimental visits consisting of 3 h cycling commencing after an overnight fast at an intensity equivalent to 95% GET. During the trials they received carbohydrates at an average rate of 120 or 90 g h^−1^ in 0.8:1 or 1:2 fructose-maltodextrin ratio, respectively. Carbohydrates were naturally high or low in ^13^C stable isotopes enabling subsequent calculations of exogenous and endogenous carbohydrate oxidation rates.

**Results:**

Exogenous carbohydrate oxidation rates were higher in the 120 g h^−1^ condition (120–180 min: 1.51 ± 0.22 g min^−1^) as compared to the 90 g h^−1^ condition (1.29 ± 0.16 g min^−1^; *p* = 0.026). Endogenous carbohydrate oxidation rates did not differ between conditions (2.15 ± 0.30 and 2.20 ± 0.33 g min^−1^ for 120 and 90 g h^−1^ conditions, respectively; *p* = 0.786).

**Conclusions:**

The results suggest that carbohydrate ingestion at 120 g h^−1^ in 0.8:1 fructose-maltodextrin ratio as compared with 90 g h^−1^ in 1:2 ratio offers higher exogenous carbohydrate oxidation rates but no additional sparing of endogenous carbohydrates. Further studies should investigate potential performance effects of such carbohydrate ingestion strategies.

## Introduction

Maintaining carbohydrate (CHO) availability during prolonged endurance exercise plays an important role as depletion of muscle glycogen stores and/or a reduction in blood glucose availability can contribute to the development of fatigue (Bergström et al. 1967; Christensen and Hansen 1939). As endogenous CHO stores are often not sufficient to sustain the required exercise intensity of a given duration, there is no surprise to see that CHO provision during exercise can improve exercise capacity and/or performance (Stellingwerff and Cox 2014) by maintaining sufficient CHO availability (Coyle et al. 1983; Hawley and Leckey 2015). Finding optimal CHO ingestion rates is especially important in sports like professional cycling where exercise intensities can be very high and exercise energy requirements such and carbohydrate that depletion of endogenously stored CHO could limit performance (Plasqui et al. 2019; Sanders and Heijboer 2019). To overcome this, athletes are advised to sustain high CHO availability by ingesting 60–90 g h^−1^ of CHO during prolonged endurance exercise lasting > 2.5 h (Burke et al. 2011; Thomas et al. 2016).

At these CHO ingestion rates, CHO have been recommended to consist of a combination of fructose- and glucose-based CHO and most studies so far investigated a 1:2 fructose to glucose ratio (Jeukendrup 2011; Wallis et al. 2005). Combining these two monosaccharide types has been shown to increase the oxidation of ingested CHO, most likely due to improved absorption from the intestines as fructose and glucose utilise different transporters (Jeukendrup and Jentjens 2000). In line with this, evidence demonstrates oxidation rates of ingested glucose-based CHO peak at the rate of 1–1.1 g min^−1^ but peak at around 1.5 g min^−1^ when fructose is added (Gonzalez et al. 2017; Rowlands et al. 2015; Wallis et al. 2005). More recently, different fructose to glucose ratios have been compared and it was found that at a fructose to glucose ratio closer to unity (i.e., 0.8:1) as compared to the established 1:2 offers benefits from efficiency of utilisation, gastrointestinal comfort, and performance standpoints, which is most likely due to more efficient intestinal absorption of both monosaccharides (O’Brien et al. 2013; O’Brien and Rowlands 2011; Rowlands et al. 2015).

When performance was assessed using carbohydrate ingestion rates ranging between 10 and 120 g h^−1^ the recommended CHO intake ingestion (i.e., 60–90 g h^−1^) was shown to be most beneficial to performance (Smith et al. 2013) despite the existence of evidence that higher exogenous oxidation rates of CHO could be achieved with higher CHO intakes (Jentjens et al. 2004; Jentjens and Jeukendrup 2005; Rowlands et al. 2015). Likely the most important reason for supplementing exogenous CHO during exercise is a reduction in reliance of endogenous CHO stores. While evidence is not clear on whether CHO ingestion can reduce muscle glycogen utilisation, high CHO intakes can markedly reduce or even completely supress liver glycogen breakdown (Gonzalez et al. 2015; Jeukendrup et al. 1999). However, as muscle glucose uptake progressively increases during prolonged exercise to compensate for a reduction in muscle glycogen content (Kristiansen et al. 1997; Richter and Hargreaves, 2013), at some point ingested and absorbed CHO during exercise become insufficient to prevent liver glycogenolysis (Jeukendrup et al. 2006). Thus, during prolonged events even in the presence of relatively high CHO ingestion rates, liver glycogen depletion can occur. CHO intake during exercise can therefore increase carbohydrate availability and thus exercise capacity, independently from sparing of muscle glycogen stores (Coyle et al. 1986; Coyle et al. 1983). However, energy, and consequently CHO, demands in elite male athletes are arguably higher than those observed in studies conducted on well-trained athletes and thus optimal CHO intake rates could potentially be higher. This notion is based on the evidence indicating that exogenous CHO oxidation rates also rise with increased exercise intensity (Pirnay et al. 1982), creating an argument that elite athletes could have benefit from higher exogenous CHO availability. Indeed, elite athletes have been observed to ingest CHO at rates higher than the recommended 90 g h^−1^ (Saris et al. 1989).

While research shows that substantially higher ingestion rates than 90 g h^−1^ are possible to be achieved without substantial gastrointestinal distress and high exogenous CHO oxidation rates can be achieved (Hearris et al. 2022; Jentjens et al. 2004; Jentjens and Jeukendrup 2005; Wallis et al. 2005), it has not been to-date established whether higher intakes than currently recommended result in lower reliance on endogenous CHO which could ultimately positively affect endurance performance. Thus, the aim of the present study was to compare exogenous and endogenous CHO oxidation rates after ingesting CHO at the widely recommended rate of 90 g h^−1^ in 1:2 (Burke et al. 2011) with the rate of 120 g h^−1^ in 0.8:1 (fructose to glucose-based CHO ratio) as has been more recently advanced (Hearris et al. 2022 and Rowlands et al. 2015). Importantly, the study was performed in highly trained male athletes whose energy turnover rates and CHO demands are such that additional CHO intake could be beneficial. We hypothesised that ingesting CHO at the rate of 120 g h^−1^ as compared to 90 g h^−1^ would result in higher: (a) exogenous, and (b) lower endogenous CHO utilisation rates while not negatively affecting gut comfort.

## Methods

### Participants

Eleven men (mean ± SD: age 33 ± 6 years, body mass 79.1 ± 9.2 kg, height 182 ± 6 cm, V̇O_2peak_ 4.86 ± 0.34 L min^−1^ (62.6 ± 7 mL kg^−1^ min^−1^), intensity corresponding to the gas exchange threshold (GET) 270 ± 17 W, intensity corresponding to the respiratory compensation point (RCP) 328 ± 32 W completed the study.

A power calculation (G*Power, version 3.1) was performed to determine the sample size and was based on a previous study in which the authors looked at varying fructose to glucose ratios (among others 0.8:1 and 1:2) with fixed glucose ingestion rate of 0.6 g min^−1^ (Rowlands et al. 2008). Exogenous CHO oxidation rates at the last time point in 0.8:1 and 1:2 fructose to glucose ratios conditions (0.71 ± 0.08 and 0.82 ± 0.13 g min^−1^, respectively) was used to compute the effect size (dz = 1.018) and it was calculated that a sample size of 10 would provide an ⍺-value of 0.05 and a power of 0.80. The main recruitment criteria for taking part in the study was GET of at least 250 W achieved during the preliminary testing visit so that it was aligned with professional cyclists’ characteristics (Sanders and Heijboer 2019). Before starting the study, participants gave informed written consent after being explained the purpose, risks, and practical details of the study. The study’s procedures were approved by the Committee of the Republic of Slovenia for Medical Ethics and the study was conducted in accordance with the Declaration of Helsinki.

### Experimental design

Participants completed 5 visits to the laboratory, including a preliminary testing visit and four experimental trials, the latter ones being separated by at least 3 days (range 3–14 days). On the day of the experimental trial, participants entered the laboratory after an overnight fast and cycled on a cycling ergometer for 180 min at the intensity equivalent to 95% of GET while ingesting one of four beverages at a CHO ingestion rate of 90 or 120 g h^−1^ and in fructose to maltodextrin ratios 1:2 or 0.8:1, respectively. During the first two sessions, participants received CHO with a low natural abundance of ^13^C and during the last two sessions of high ^13^C abundance to be able to accurately quantify exogenous CHO oxidation rates. The order of both trials was randomised using an online research randomiser tool (https://www.randomizer.org). Fingertip capillary blood and expired breath samples were collected/analysed continuously throughout the trial for the assessment of whole-body metabolism.

### Preliminary testing

Participants performed two exercise bouts to accurately determine V̇O_2peak_ and the intensities corresponding to GET and the RCP as per a previously described protocol (Iannetta et al. 2020). In brief, the test started with a 2-min warm-up at 80 W followed by 6-min cycling at 120 W (moderate-intensity exercise domain). This transitioned into a ramp incremental protocol increasing the exercise intensity by 30 W min^−1^ until task failure. After 30 min of passive rest, participants cycled for 12 min at 50–65% of maximal power output achieved during the ramp incremental protocol (i.e., cycling in the heavy intensity exercise domain).

During the test, gas exchange measurements were performed using an automated online gas analysis system (MetaLyzer 3B-R3, Cortex, Lepizig, Germany). Prior to each trial, gas analysers were calibrated with a known gas mixture (15.10% O_2_, 5.06% CO_2_; Linde Gas, Prague, Czech Republic) and the volume transducer was calibrated with a 3-L calibration syringe (Cortex, Leipzig, Germany). During all tests, the participants used their own bicycles mounted onto an electrically braked cycle ergometer (Kickr V5, Wahoo, Atlanta, Georgia, USA). V̇O_2peak_ was considered to represent the highest 30-s average of O_2_ uptake.

From the obtained data, exercise intensities corresponding to the GET (i.e., the boundary between moderate and heavy exercise intensity domain) and RCP (i.e., the boundary between heavy and severe exercise intensity domain) were determined as previously described (Iannetta et al. 2020). In brief, ramp test respiratory data were analysed by two experienced researchers that independently determined oxygen uptake associated with GET (V̇O_2_ at which V̇CO_2_ and ventilation began to increase disproportionately in relation to V̇O_2_) and RCP (V̇O_2_ at which end-tidal PCO_2_ began to fall after a period of isocapnia). Subsequently, a spreadsheet supplementing the original article describing the protocol (http://links.lww.com/MSS/B957) was used to determine exercise intensities relating to GET and RCP taking into account the delay in oxygen uptake for a given power output.

### Pre-experimental control

Participants were given a list of foods known to be naturally abundant in ^13^C and asked to avoid those for 5 days before each experimental trial to minimise the background shift from glycogen stores. In addition to this, they were asked to replicate the diet and activity for 2 days preceding each experimental trial.

### Experimental trials

Participants attended the laboratory in an overnight fasted state between 6:00 and 9:00 AM. Resting expired breath samples were collected into 10-mL evacuated tubes (Exetainer Breath Vial, Labco Ltd.; Buckinghamshire, UK) and were filled from a mixing chamber to subsequently determine the ^13^C/^12^C ratio at rest and every 30 min during a 180-min long exercise bout. The exercise consisted of 180 min of cycling at 95% GET (mean 256 ± 16 W, range 235–295 W). Respiratory gas exchange measurements V̇O_2_ and V̇CO_2_ were measured every 30 min during exercise and capillary blood was obtained for assessment of blood glucose (Accu-Chek Aviva, Roche, Switzerland) and blood lactate (Lactate Plus, Nova Biomedical, USA). Every 15-min, heart rate (HR) was measured, rate of perceived exertion (RPE) was evaluated (Borg 1982) and gastrointestinal comfort (GC) was assessed by a 10-point Likert scale looking at nausea, stomach fullness, and abdominal cramping (Thorburn et al. 2006).

### Test beverages

Participants ingested a total of 2.3 L of a test beverage in each trial containing either 270 g of fructose and maltodextrin (1:2 ratio) or 360 g of fructose and maltodextrin (0.8:1 ratio) to deliver CHO at an average rate of either 1.5 g min^−1^ (90 g h^−1^) or 2 g min^−1^ (120 g h^−1^). A 500-mL bolus was provided at the exercise onset followed by 150-mL drinks every 15 min. To allow quantification of exogenous CHO oxidation rates, CHO were selected to have either a high or a low natural abundance of ^13^C [Maltodextrin High − 11.64 and − 11.31 δ‰ vs Pee Dee Bellemnitella (PBD) (Bulk, Sports Supplements Ltd., Essex, UK); Maltodextrin Low − 26.73 δ‰ vs PBD (Avebe, Veendam, The Netherlands), Fructose High − 13.11 δ‰ vs PBD (Peak Supps, Bridgend, UK), Fructose Low − 26.25 δ‰ vs PBD (Peak Supps, Bridgend, UK)]. Low ^13^C trials were used exclusively to quantify the background shift in breath ^13^CO_2_ to allow for correction and more accurate quantification of exogenous CHO oxidation rates (Odell et al. 2020).

### Breath analyses

Isotopic enrichment of breath samples was determined using gas chromatography isotope ratio mass spectrometry (Europa Scientific Hydra 20-20, Crewe, United Kingdom). ^13^C enrichment of ingested CHO was determined by elemental analyser isotope ratio mass spectrometry (Europa Scientific Hydra 20-20).

### Calculations

Total CHO and fat oxidation rates were calculated using recommended equations (Jeukendrup and Wallis 2005) assuming protein oxidation to be negligible:

$${\text{Total\,carbohydrate\,oxidation}}\,\left[ {\text{g}} \, {\text{min}} ^{ - 1}  \right] = \,4.210 \cdot \mathop {\text{V}}\limits^{.} {\text{CO}}_{2} - 2.962 \cdot \mathop {{\text{VO}}_{2} }\limits^{.}$$

$$\text{Total\,fat\,oxidation} \left[  {\text{g}} \,{\text{min}} ^{ - 1} \right]{\mkern 1mu} \,=1.695\cdot \dot{\text{V}}{{\text{O}}}_{2}-1.701\cdot \dot{\text{VC}}{{\text{O}}}_{2}$$

In which V̇O_2_ and V̇CO_2_ are measured in litres per minute.

The isotopic enrichment of expired breath samples was expressed as δ per millilitre difference between the ^13^C/^12^C of the sample and a known laboratory reference standard using an established equation (Mosora et al. 1976). δ^13^C was then related to an international standard Pee Dee Bellemnitella (PDB).

Exogenous CHO oxidation rates were subsequently calculated using the following equation (Péronnet et al. 1993):

$$\text{Exogenous carbohydrate oxidation} \left[{\text{g}} \, {\text{min}}^{-1}\right]=\dot{\text{VC}}{{\text{O}}}_{2}\cdot \frac{\mathrm{\delta Exp}-\updelta {\text{Exp}}_{\text{bkg}}}{\mathrm{\delta Ing}-\updelta {\text{Ing}}_{\text{bkg}}}\cdot \frac{1}{k}$$

where δExp is the ^13^C enrichment of the expired air at various time points in the High ^13^C trials, δIng is the enrichment of the ingested beverage, δExp_bkg_ is the ^13^C enrichment of the expired air at the corresponding time points in the low ^13^C trial, δIng_bkg_ is the ^13^C enrichment of the carbohydrate drink in Low ^13^C trials, and *k* is the amount of CO_2_ (in L) produced by the complete oxidation of 1 g of glucose (*k* = 0.7467 L). Exogenous CHO oxidation rates were analysed for the whole duration of the study as 30 min is sufficient for the bicarbonate pool to turn over (Podlogar and Wallis 2020).

The oxidation efficiency was determined as the fraction of the ingested CHO that were oxidised in the last 60 min (120–180 min) as compared to the 90 or 120 g h^−1^ ingestion rate during the whole exercise bout.

### Statistics

Data were initially tested for sphericity using Mauchly’s test. Then, a two-way ANOVA for repeated measures was used to compare differences between conditions. When necessary, analyses were adjusted using the Greenhouse–Geisser correction. Where significant effects were observed by ANOVA for time × condition interaction, *post-hoc* comparisons were made with paired *t*-tests with the Tukey test applied to account for multiple comparisons. All values are presented as mean ± SD. Statistical significance was set at *p* < 0.05. Data analysis was performed using Prism (Version 9; GraphPad Software, San Diego, CA, US).

## Results

### Stable isotope measurements

**Fig. 1:**
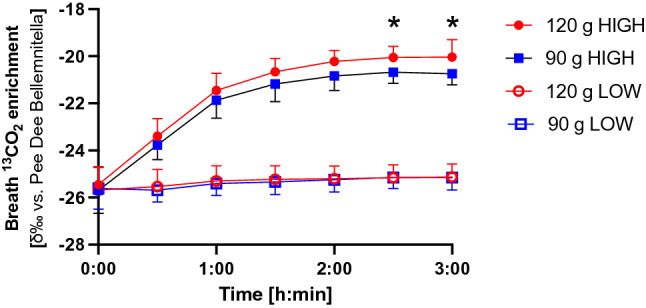
Breath ^13^CO_2_ enrichment during exercise with ingestion of CHO at different rates (90 and 120 g h^−1^) and different ^13^C natural enrichment (low or high). *Denotes significant difference between 120 g HIGH and 90 g HIGH

Resting breath ^13^C enrichment was similar at the beginning of all four experimental trials, averaging − 25.68 ± 0.963, − 25.61 ± 0.88, − 25.45 ± 0.77 and − 25.70 ± 0.98 δ‰ vs PDB for 120 g Low ^13^C, 90 g Low ^13^C, 120 g High ^13^C and 90 g High ^13^C, respectively. Expired breath ^13^C is shown in Fig. 1. There were significant differences over time, between conditions, and a significant time × condition interaction (all *p* < 0.001) so that both HIGH trials differed from LOW trials from 30-min time points. 120 g HIGH and 90 g LOW differed at 2h 30 min and 3 h time points (*p* = 0.024 and 0.033, respectively).

### Substrate oxidation rates and blood metabolites

**Fig. 2:**
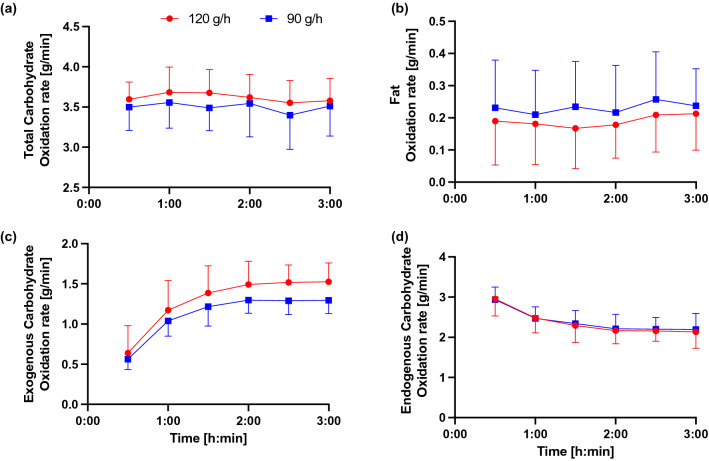
Total CHO **a** fat, **b** exogenous CHO, **c** endogenous CHO and **d** oxidation rates were measured every 30 min in 120 and 90 conditions

Total CHO oxidation rates (Fig. 2a) did not change over time (*p* = 0.131) and there was no difference between the conditions (*p* = 0.167). Similarly, fat oxidation rates (Fig. 2b) did not change over time (*p* = 0.215) and did not differ between conditions (p = 0.280). Exogenous CHO oxidation rates (Fig. 2c) increased over time (*p* < 0.001) and were significantly higher in the 120 g condition as compared to 90 g (*p* = 0.026). There was no time × condition interaction (*p* = 0.205). The contribution of endogenous CHO oxidation (Fig. 2d) towards total energy turnover decreased over time (*p* < 0.001) but did not differ between the conditions (*p* = 0.786).

Peak exogenous CHO oxidation rates (i.e., highest measured value at one time point) were higher in 120 g condition as compared to 90 g (1.60 ± 0.25 vs 1.36 ± 0.17 g min^−1^ for 120 and 90 conditions, respectively; *p* = 0.005). Oxidation efficiency for the last 60 min of the experimental trials was higher in 90 g condition as compared to 120 g (86 ± 10% and 76 ± 11% for 90 and 120 conditions, respectively; *p* = 0.012).

Oxygen uptake, respiratory exchange ratio (RER), blood glucose and blood lactate results are presented in Table 1. There were no differences between conditions (*p* > 0.05) and no change over time (*p* > 0.05) in any of the parameters.

**Table 1:** Oxygen uptake, respiratory exchange ratio, blood glucose and blood lactate

Time [h:min]	Trial	VO_2_ [L min^−1^]	RER	Blood glucose [mmol L^−1^]	Blood lactate [mmol L^−1^]
0:30	120	3.30 ± 0.18	0.96 ± 0.02	5.5 ± 1.2	1.7 ± 0.4
	90	3.30 ± 0.18	0.97 ± 0.03	5.6 ± 1.4	1.7 ± 0.4
1:00	120	3.35 ± 0.17	0.96 ± 0.02	5.3 ± 0.8	1.8 ± 0.4
	90	3.30 ± 0.21	0.96 ± 0.02	5.4 ± 1.1	1.6 ± 0.6
1:30	120	3.32 ± 0.19	0.97 ± 0.02	5.1 ± 0.8	1.6 ± 0.5
	90	3.30 ± 0.16	0.96 ± 0.02	5.6 ± 1.0	1.6 ± 0.5
2:00	120	3.29 ± 0.18	0.96 ± 0.02	5.7 ± 0.7	1.6 ± 0.3
	90	3.31 ± 0.17	0.96 ± 0.03	5.0 ± 0.8	1.5 ± 0.6
2:30	120	3.30 ± 0.17	0.96 ± 0.02	5.1 ± 0.8	1.7 ± 1.0
	90	3.27 ± 0.16	0.95 ± 0.03	5.3 ± 0.7	1.5 ± 0.4
3:00	120	3.33 ± 0.18	0.96 ± 0.02	5.2 ± 0.7	1.3 ± 0.3
	90	3.32 ± 0.17	0.95 ± 0.02	4.8 ± 0.6	1.3 ± 0.3

*RER* respiratory exchange ratio

### RPE and GI comfort

**Fig. 3:**
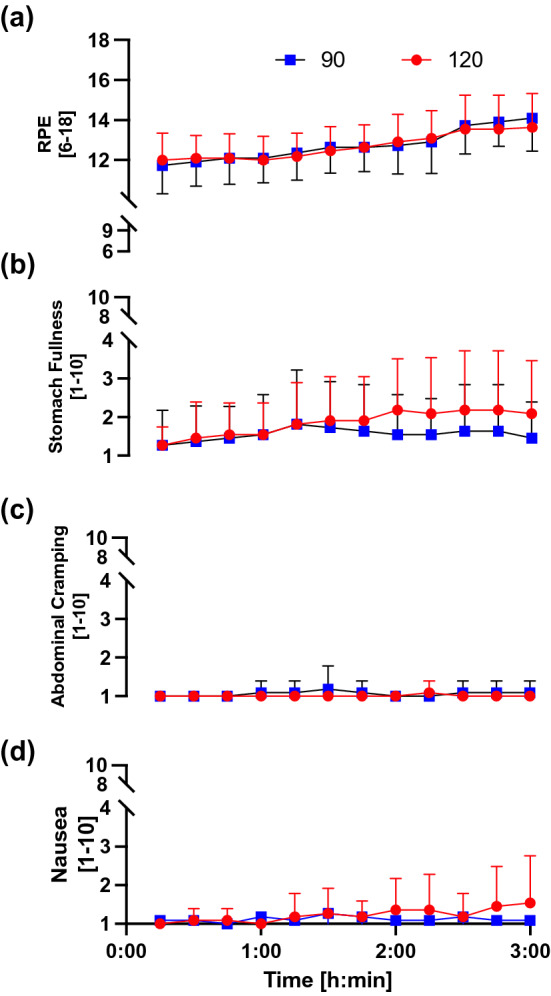
Rate of perceived exertion (RPE) **a** stomach fullness, **b** abdominal cramping, **c** nausea, and **d** during experimental trials assessed every 15 min

RPE and ratings of GI comfort including stomach fullness, abdominal cramping and nausea are presented in Fig. 3. There were no significant differences between conditions in any of the measures (*p* > 0.05). RPE increased over time (*p* < 0.001), whereas stomach fullness, abdominal cramping and nausea remained unchanged over time (*p* = 0.067, 0.341 and 0.249; respectively).

## Discussion

The results of the present study confirm the first hypothesis that ingesting CHO during prolonged endurance exercise in highly trained cyclists at a rate of 120 g h^−1^ with a 0.8:1 fructose to maltodextrin ratio as compared to the currently recommended 90 g h^−1^ with a 1:2 ratio results in higher exogenous CHO oxidation rates, without significant gastrointestinal discomfort. However, the results reject the second hypothesis as higher exogenous CHO oxidation rates with 120 g h^−1^ with a 0.8:1 fructose to maltodextrin ratio as compared to the currently recommended 90 g h^−1^ with a 1:2 ratio did not translate into a further reduced utilisation of endogenous CHO stores.

The primary aim of CHO ingestion during prolonged endurance exercise is to a) reduce reliance on endogenous CHO stores and/or b) maintain sufficient CHO availability by the provision of exogenous CHO (Coyle et al. 1986; Jeukendrup and Jentjens 2000). Indeed, studies have demonstrated sparing of endogenous CHO stores by ingestion of multiple transportable CHO (i.e., fructose and glucose) as compared to glucose-based CHO only at an ingestion rate of > 1 g min^−1^ due to increased reliance on exogenous CHO (Rowlands et al. 2015; Wallis et al. 2005). There appears to be a curvilinear dose–response relationship between ingestion of multiple transportable CHO and exogenous CHO oxidation rates with exogenous CHO oxidation rates peaking just above 1.5 g min^−1^ or 90 g h^−1^ even when CHO intake is higher (Gonzalez et al. 2017; Rowlands et al. 2015). A reduction in efficiency of oxidation of ingested CHO with high doses has usually been attributed to metabolic properties of fructose as peak exogenous glucose oxidation rates are known to be 1–1.1 g min^−1^ even in the presence of higher glucose intake and are likely limited by an intestinal transport (Jeukendrup and Jentjens 2000). The observation that the oxidation efficiency of ingested fructose is lower is likely a result of either lower intestinal transport or the requirement for fructose to be first converted to glucose or lactate in the splanchnic tissue before being readily available to the skeletal muscle for oxidation (Lecoultre et al. 2010; Rowlands et al. 2015).

In the present study, a different fructose to maltodextrin ratio between conditions was chosen for the following reasons. At 90 g h^−1^ the 1:2 ratio was used as the glucose component is at or near the intestinal transport limitation (Jeukendrup and Jentjens 2000), and because this ratio has been used in studies constituting the current CHO intake guidelines (Burke et al. 2011; Thomas et al. 2016). The 0.8:1 ratio in 120 g h^−1^ on the other hand was chosen as more recent evidence indicates that this is a superior ratio resulting in higher CHO oxidation efficiency and lower prevalence of GI discomfort as compared to the 2:1 ratio (O’Brien et al. 2013; O’Brien and Rowlands 2011; Rowlands et al. 2015). This dose (i.e., 120 g h^−1^) at this ratio has recently been investigated in a study comparing different CHO formats (i.e., drink, gel, chew, and mix) and exogenous CHO oxidation rates higher than 1.5 g min^−1^ were reported; however, the rates have not been compared with other doses (e.g., 90 g h^−1^) (Hearris et al. 2022). Results presented in the current study support the notion of diminished return with higher CHO intake rates, as oxidation efficiency was higher in the 90 g condition compared to the 120 g condition. This could most likely be attributed to a difference in fructose oxidation as the difference in ingestion of glucose-based CHO was only 6 g h^−1^ (60 vs. 67 g h^−1^).

However, when interpreting exogenous CHO oxidation rates, one also needs to consider the actual energy and CHO demands during exercise as those also appear to have a role in exogenous CHO oxidation rates (Massicotte et al. 1994; Pirnay et al. 1982). For instance, studies by Jentjens and colleagues that investigated exogenous CHO oxidation rates when ingesting CHO at a rate of 2.4 g min^−1^ (144 g h^−1^) (Jentjens et al. 2004; Jentjens and Jeukendrup 2005) also had a condition in which only water was provided. In the water condition, total CHO oxidation rates were almost 1 g min^−1^ lower than the CHO ingestion rate during exercise when CHO were provided at the rate of 2.4 g h^−1^. While ingestion of CHO at this rate ultimately reduced the endogenous CHO oxidation rates, one could also hypothesise that the utilisation of exogenous CHO oxidation was not maximal due to insufficient CHO demand. Provided that CHO were absorbed into the bloodstream, some CHO could have been directed towards storage in non-active tissue rather than oxidation. Based on this, in the present study, we recruited participants whose training status allowed them to comfortably exercise at higher power outputs and by doing that achieve greater energy turnover rates and higher CHO demands during exercise.

As expected, based on high CHO demands and high CHO ingestion rates, we observed very high absolute exogenous CHO oxidation rates averaging 1.51 ± 0.22 g min^−1^ for the last 60-min of exercise with the ingestion rate of 120 g h^−1^, with 2 participants reaching peak exogenous CHO oxidation rates of 2.0 g min^−1^. While individual peak exogenous CHO oxidation rates are higher than the previously reported 1.86 g min^−1^ by Hearris and colleagues (2022) and 1.75 g min^−1^ reported by Jentjens and Jeukendrup (2005), average exogenous CHO oxidation rates in the last 60 min (~ 1.51 g min^−1^) are consistent with the data by Hearris and colleagues that reported oxidation of ~ 1.56 g min^−1^ at a lower power output (~ 216 W vs. present study at ~ 256 W) but lower than in studies by Jentjens and colleagues (2004) and Jentjens and Jeukendrup (2005) where oxidation rates of ~ 1.69 and 1.67 g min^−1^ were reported at the mean power out of 179 W and 188 W, respectively. Of note is that exogenous CHO oxidation rates may be overestimated in studies by Jentjens and colleagues due to methodological issues with using water as a background trial for determination of exogenous CHO oxidation rates (Odell et al. 2020). Collectively, the results of these three previous studies, and the present data, where CHO ingestion was provided at a rate of ≥ 120 g h^−1^, do not show a relationship between absolute exercise intensity and exogenous CHO oxidation rates, which contrasts previous work (Massicotte et al. 1994; Pirnay et al. 1982). While it is difficult to directly compare the study results directly due to methodological differences, discrepancy in the findings of whether metabolic rate has an impact on exogenous CHO oxidation rates, calls for more research in the area to better understand this issue.

Despite higher exogenous CHO oxidation rates, there was no sparing of endogenous CHO stores with an ingestion rate of 120 g h^−1^ as compared with 90 g h^−1^. While perhaps counterintuitive, increasing exogenous carbohydrate oxidation rates by hyperglycaemia also did not further reduce endogenous carbohydrate use (Hawley et al. 1994). In addition to this, some previous studies even demonstrated higher estimated muscle glycogen utilisation with high CHO intakes during exercise (King et al. 2018; Wallis et al. 2007), the mechanisms for which remain to be elucidated. One of the proposed mechanisms was that the gut discomfort and stress associated with higher intakes could increase total CHO oxidation rates (Wallis et al. 2007). However, lack of differences in gastrointestinal discomfort between both conditions in the present study discounts this option. Based on the measures collected in this study, it is difficult to speculate why there were differences in exogenous but not endogenous CHO oxidation rates. Unfortunately, lack of blood plasma/serum storage in the present study did not enable investigation of differences in hormones (e.g., insulin) and/or other metabolites (e.g., non-esterified fatty acids and glycerol) that would ultimately provide more insights into the effects on metabolism. Absence of differences in endogenous CHO utilisation rates at an average exercise intensity that is comparable to those observed in professional cycling (Sanders and Heijboer 2019) casts a doubt whether there are any functional benefits of ingesting CHO at rates higher than 90 g h^−1^.

Future studies should explore whether there would have been any performance benefits of ingesting higher amounts of CHO during exercise, preferably by simulating a prolonged endurance event where power output and thus energy requirements are not constant but variable such as triathlon or cycling where ingestion rates higher than 90 g h^−1^ have already been reported (Pfeiffer et al. 2012). Speculatively, a higher CHO availability caused by a higher exogenous CHO availability could play a beneficial role at higher exercise intensities (i.e., in heavy and severe exercise intensity domains). There, reliance on CHO oxidation is highest and perhaps ingestion rates > 90 g h^−1^ could have resulted in better performance when endogenous CHO availability is insufficient as previous evidence indicates that once skeletal muscle glycogen levels are low, blood derived CHO sources start to play a decisive role (Coyle et al. 1986). In addition to this, a relatively large interindividual variability in maximal exogenous CHO oxidation rates observed in the present and in previous studies (Hearris et al. 2022) calls for further work on what determines maximal exogenous CHO oxidation rates and whether there is a potential to optimise CHO availability by individually prescribing CHO intakes during exercise. Also, it remains to be explored if the data from present study can be extrapolated to both sexes. Direct assessment of liver and muscle glycogen utilisation would also clarify if endogenous carbohydrate utilisation in those depots was differentially affected in response to varying ingested carbohydrate dose during exercise.

In conclusion, this study demonstrates that by ingesting high amounts (i.e., 120 g h^−1^) of multiple transportable CHO (i.e., fructose and maltodextrin in 0.8:1 ratio) exogenous CHO oxidation rates are ~ 17% higher as compared to when CHO ingestion rate is lower (i.e., 90 g h^−1^). However, higher exogenous CHO oxidation rates did not translate into sparing of endogenous CHO utilisation. From a whole-body metabolism point of view, this study’s data demonstrate that ingesting CHO at a higher rate than currently recommended 90 g h^−1^ does not provide any benefits.
